# Effect of commercial slow-release urea product on in vitro rumen fermentation and ruminal microbial community using RUSITEC technique

**DOI:** 10.1186/s40104-022-00700-8

**Published:** 2022-05-06

**Authors:** Yongmei Guo, Ling Xiao, Long Jin, Sumei Yan, Dongyan Niu, Wenzhu Yang

**Affiliations:** 1 Inner Mongolia Key Laboratory of Animal Nutrition and Feed Science, College of Animal Science, Inner Mongolia Agricultural University, Hohhot, 010018 Inner Mongolia China; 2 Agriculture and Agri-Food of Canada, Lethbridge Research and Development Centre, Lethbridge, AB T1J 4B1 Canada; 3 Hangzhou King Techina Feed Co., Ltd, Hangzhou City, China; 4 College of Veterinary Medicine, University of Calgary, 2500 University Dr. NW, Calgary, AB T2N 1N4 Canada

**Keywords:** Artificial rumen system, Dairy cow diet, Fermentation characteristics, Slow-release urea

## Abstract

**Background:**

The objectives of this study were to determine the effect of commercial slow-release urea (SRU) on in vitro fermentation characteristics, nutrient digestibility, gas production, microbial protein synthesis and bacterial community using a rumen simulation technique (RUSITEC). The experiment was a completely randomized design with four treatments and four replications of each treatment. Treatments were: control diet (no SRU addition), control diet plus 0.28% SRU (U28), or plus 0.56% SRU (U56), and control diet that was modified substituting a part of soybean meal equivalent to 0.35% SRU (MU35; dry matter [DM] basis). The experiment consisted of 8 d of adaptation and 7 d of data and sample collection. Rumen inoculum was obtained from three ruminally fistulated Angus cows fed the same diet to the substrate incubated.

**Results:**

Digestibility of DM, organic matter (OM), crude protein (CP), fibre and starch was not affected, but daily production of gas (*P* < 0.07) and methane (*P* < 0.05) was quadratically increased with increasing SRU supplementation. The increase of SRU addition did not affect fermentation pH and total volatile fatty acid (VFA) production, whereas linearly (*P* < 0.01) decreased proportion of propionate, and linearly (*P* < 0.01) increased acetate to propionate ratio and ammonia nitrogen (N) concentration. The microbial N efficiency was also linearly (*P* < 0.03) improved with increasing supplementation of SRU. In comparison with control diet, the dietary substitution of SRU for part of soybean meal increased (*P* < 0.05) the digestibility of DM, OM and CP and decreased (*P* < 0.02) the total gas production. The total VFA production and acetate to propionate ratio did not differ between control and MU35, whereas the proportion of butyrate was lower (*P* < 0.05) and that of branched-chain VFA was greater (*P* < 0.05) with MU35 than control diet. Total and liquid-associated microbial N production as well as ammonia N concentration were greater (*P* < 0.03) with MU35 than control diet. Observed operational taxonomic units (OTUs), Shannon diversity index, and beta diversity of the microbial community did not differ among treatments. Taxonomic analysis revealed no effect of adding SRU on the relative abundance of bacteria at the phylum level, while at the genus level, the beneficial impact of SRU on relative abundance of *Rikenellaceae* and *Prevotellaceae* in feed particle-associated bacteria, and the abundance of *Roseburia* in liquid associate bacteria was greater (*P* < 0.05) with MU35.

**Conclusions:**

Supplementation of a dairy cow diet with SRU showed potential of increase in ammonia N concentration and microbial protein production, and change fermentation pattern to more acetate production. Adding SRU in dairy cow diet also showed beneficial effect on improving digestibility of OM and fibre. The results suggest that SRU can partially substitute soybean meal in dairy cow diet to increase microbial protein production without impairing rumen fermentation.

## Background

Dietary protein plays an important role in ruminant nutrition for providing amino acids and the nitrogen (N) source for microbial protein production in the rumen. However, dietary protein often comes from expensive feeds, such as soybean meal, and thus a strategy to reduce feed cost without negatively impact on animal production needs to be considered. Non-protein nitrogen (NPN), such as urea, is commonly used as a replacement of feed protein in ruminant diets due to its low cost [1]. Whereas, the urea could be poorly utilized as a N source for microbial protein synthesis because it is rapidly hydrolyzed by rumen bacterial ureases to ammonia, and it often exceeds the use capacity by rumen bacteria with the surplus being absorbed through the rumen wall into the blood [2]. Synchronizing N supply with microbial requirements in the rumen is an important nutritional strategy to improve energy and N utilization. A way to improve the N synchronization with microbial need is to slow down the release rate of urea into ammonia by coating the granules with substrates that is able to protect them from rapid degradation [3]. To achieve this goal, numbers of rumen protected urea products, such as oil-coated, calcium-bound, polymer-coated sources of urea have been developed [4]. Although numerous studies have tested the effectiveness of slow-release urea (SRU) product in ruminant diets to improve ammonia assimilation in the rumen [5–8], the results were substantially variable. The behavior of SRU likely depends on the rate of urea release, amount of inclusion, type of diets, the nature of the rumen microbiota, and host traits, such as absorption of ruminal NH_3_ and liquid and solid passage rates.

Although the use of SRU is primarily to provide a steady supply of N to synchronize energy availability for supporting the microbial protein synthesis, adding the SRU in the ruminant diets appeared not only for providing N source in the rumen. Supplementation of SRU also affected rumen microbial diversity and community composition. Alipour et al. [9] suggested an improvement of fibrolytic bacterial activity by adding SRU in a high-grain diet. Ferme et al. [10] reported an inhibition of major ammonia producing bacteria (e.g., *Prevotella ruminantium *and* Prevotella bryantii*) and thus a reduction in ammonia N (NH_3_-N) concentration in continuous culture. Moreover, several studies reported that the lower dose of SRU actually resulted in better growth performance of yak fed diet supplemented with 1% than with 2% of SRU [8] or the higher fibre digestibility of a high-grain diet with 0.5% and 1% SRU than 1.75% SRU supplementation in Rusitec [9]. For lactating dairy cows, Grossi et al. reported that inclusion of 1.33% SRU to partly replacing feed protein in dairy cow diet improved feed intake, milk yield and production efficiency as well as reduced methane production and carbon footprint due to SRU feeding [11]. In a meta-analysis, Salami et al. found that supplementation of dairy cow diets with SRU was beneficial to improve feed efficiency and to decrease carbon footprint and manure nitrogen excretion but limited effect on milk production [12]. These reports highlight particularly the beneficial impact on feed efficiency and environmental sustainability. Salami et al. suggested that the improved feed efficiency by adding SRU may be associated with enhanced N capture and modulation of rumen microbiome [12]. However, the information on the effect of SRU on rumen microbiome is scarce in literature, and with our best knowledge, only one study demonstrated significant influence of rumen microbiota but in yak by feeding SRU.

In addition, the soybean meal (SBM) is one of the commonly used as a prominent source of protein in dairy cow diets. It offers a good balance and availability of amino acids, and its amino acid profile is similar with that of ruminal bacteria. However, feeding SBM to dairy cows had a higher carbon footprint than SRU feeding, and thus increased the environmental load [12]. Additionally, due to the fluctuation in price of SBM and persistent increase in recent years, searching in alternatives of SBM comes to an inevitable trend to reduce production costs, maintain or maximize cattle performance and decrease greenhouse gas emission resulting from the excretion of N [13]. Therefore, the use of NPN replacement is attractive in cattle diets because of its low cost [7, 14]. However, the consequences of replacing sources of true protein with SRU in diets remain unclear. We hypothesized that rumen fermentation characteristics and microbial community composition would be affected with increasing addition of SRU or partly substitution of SBM with SRU in dairy cow diet because of its improvement of microbial N utilization through slow ammonia release and higher microbial synthetic efficiency in the rumen. The objectives of this study were to evaluate the effects of 1) increasing dosages of SRU supplementation and 2) substituting a part of soybean meal equivalent to 0.35% SRU in a forage-based diet on rumen fermentation, nutrient digestibility, microbial protein synthesis and microbial community in Rusitec.

## Methods

### Experimental design

The experiment was a completely randomized design with four treatments assigned to sixteen fermentation vessels in two eight-vessel RUSITEC apparatus with four replications for each treatment. The treatments were control diet (no SRU), control plus 0.28% SRU (U28), control plus 0.56% SRU (U56), and control diet that was modified substituting a part of soybean meal equivalent to 0.35% SRU. The diets were formulated to be isoenergetic, but protein concentration of U28 and U56 increased due to SRU addition and isonitrogenic between control and U35 diets (Table 1). The diets of control, U28 and U56 were kept as the same formulation except that the SRU was additional to avoid confounding between the formulation and SRU treatments. The SRU product was provided by King Techina Feed Co., Ltd. (Hangzhou, China) and it was prepared based on a matrix of urea pills (87% urea or 40.4% N, DM basis) and palm oil and sodium carboxymethyl cellulose (13%). The release rate of SRU was 52.1% at 6 h and 71.3% at 12 h of incubation in artificial rumen fluid. The experiment was conducted as one period consisting of 15 d, including 8 d of adaptation and followed by 7 d for sample and data collection. The basal diet was prepared in the form of total mixed ration (TMR), and ground through a 4-mm sieve. Approximately 10 g (dry matter, DM) of the basal diet was weighed into nylon bags (10 cm × 20 cm; pore size of 50 μm, Ankom Technology Corp., Macedon, NY, USA), and SRU was added to bags at the desired concentration followed by manual mix.

**Table 1:** Ingredient and chemical composition of experimental diets

Item	Diets			
	Control	U28	U56	MU35
Ingredient, %				
Corn silage	27.1	27.1	27.1	27.1
Alfalfa hay	11.4	11.4	11.4	11.4
Ort hay	7.0	7.0	7.0	7.0
Steam flaked corn	24.4	24.4	24.4	26.1
Soybean meal, (46%)	10.6	10.6	10.6	8.9
Canola meal	2.8	2.8	2.8	2.8
Extrude soybean	3.4	3.4	3.4	3.4
DDGS^a^	2.1	2.1	2.1	2.1
Dried beet pulp	6.84	6.84	6.84	6.84
Mineral and vitamin	1.82	1.82	1.82	1.82
Mycotoxin binder	0.08	0.08	0.08	0.08
Soda	0.57	0.57	0.57	0.57
Saturated fat	1.90	1.90	1.90	1.90
Slow-released urea^b^	0	0.28	0.56	0.35
Chemical composition, % of DM				
DM, % of fresh matter	91.2	91.2	91.2	91.3
OM	92.4	92.4	92.4	92.5
CP	16.2	16.9	17.7	16.2
NDF	30.4	30.4	30.4	30.5
ADF	19.0	19.0	19.0	19.0
Starch	27.1	27.1	27.1	28.2
NE_L_^c^, Mcal/kg	1.71	1.71	1.71	1.71

^a^DDGS = Distillers dried grains with solubles

^b^The product contains 13% of coating materials of palm oil and sodium carboxymethyl cellulose and 87% of urea

^c^NE_L_ = Net energy for lactation

### Inoculum donor

The experimental protocols were reviewed and approved by the Lethbridge Research and Development Centre Animal Care Committee, and cattle were handled in accordance with the guidelines of the Canadian Council on Animal Care [15].

Three ruminally cannulated Angus cross cows (averaging 668 ± 55.1 kg body weight) that fed a TMR similar to the diet used in Rusitec (containing 35% corn silage, 10% mixed hay, and 55% corn-based concentrate mix, DM basis) were used as inoculum donor. Two hours after morning feeding, solid rumen digesta and rumen liquid were collected from four locations within the rumen of each cow via rumen cannula. Contents were immediately pooled over in equal amount (4 L per cattle), filtered through four layers of cheesecloth into an insulated thermos and transported to the laboratory directly. Rumen liquid was well mixed, pH recorded and kept at 39 °C in a water bath prior to introduction into fermenters.

### Experimental procedure

Two RUSITEC apparatuses were used in this study with each equipped with eight 920-mL volume anaerobic fermenters, as described in previous study [16]. The fermenters were randomly chosen, and each fermenter was outfitted with a port for buffer input, and a port for effluent output. To begin the experiment, each fermenter was filled with 200 mL of McDougalls buffer (pH = 8.2) [17], 700 mL of strained rumen fluid, and two pre-labeled nylon bags with one containing 20 g of mixed solid rumen digesta from four donor cattle and one containing diet substrate with or without SRU addition. The fermenters were incubated in a water bath at 39 °C. After 24 h of incubation, the nylon bag containing solid rumen digesta was removed from fermenter and replaced by one new bag with substrate with or without SRU. Thereafter, one diet bag was replaced daily in the morning so that each bag will remain in the fermenter for 48 h except the last day when one bag in each vessel was removed after 24 h. Artificial saliva [17] was infused into the fermenter continuously by a peristaltic pump set (Model ISM 932D, Ismatec, Index Health and science GmbH, Wertheim, Germany) at a dilution rate of 2.9%/h. On d 8, the chemical composition of the artificial saliva was modified by mixing ammonium sulfate enriched with ^15^N ((^15^NH_4_)_2_SO_4_; Sigma Chemical Co., St. Louis, MO, USA; minimum ^15^N enrichment 1 g/L) into the infused buffer solution to label bacteria. Daily effluent was collected into a 2-L volumetric flask, and gas was collected in a 2-L bag (CurityR; Conviden Ltd., Mansfield, MA, USA). The effluent in each flask was preserved with 3 mL of a sodium azide solution to stop the microbial activity during the sample collection period.

### Nutrient disappearance

Nutrient disappearance including DM, organic matter (OM), crude protein (CP), acid detergent fibre (ADF), neutral detergent fibre (NDF) and starch was measured from d 9 to 13 of the sampling period [18]. Feedbags were removed from fermenters after 48-h incubation and then hand-washed under running water until the water runoff was clear. Afterwards, feedbags were dried in oven at 55 °C for 48 h for DM disappearance determination [19]. The residues in bags from the same fermenter were collected over 5 d and pooled by fermenter. Feeds and feed residues were grounded through a 1-mm screen using a Wiley mill (standard model 4 Arthur Thomas Co., Philadelphia, PA, USA) for chemical analysis. Part of grounded feeds and feed residue was further ground with a ball mill (Mixer Mill MM2000; Retsch, Haan, Germany) for total N and starch analysis. Ash content was analyzed by combustion samples at 550 °C for 5 h (method 942.05) [19], and OM content was calculated as 100 minus the ash content. The NDF and ADF were determined on a VELP Fiber Digestion System (VELP Scientifica, Burlington, ON, Canada) using the method as described by Van Soest et al. [20] and AOAC (method 973.18) [19], respectively. Total N was determined using a combustion analyzer (NA 2100, Carlo Erba Instruments, Milan, Italy) according to Smith and Tabatabai [21], and CP was calculated as total N × 6.25. Starch was determined by enzymatic hydrolysis of α-linked glucose polymers [22]. Disappearance of DM, OM, CP, NDF, ADF, and starch was calculated as the differences between the amount of individual component in substrates before incubation and that in residues after 48 h of incubation. The chemical analysis was conducted in duplicate, and repeated if the CV for the replicate analysis was more than 5%.

### Fermentation parameters, gas and methane production

Fermenter pH using a pH meter (Orion model 260A, Fisher Scientific, Toronto, Canada) and the volume of daily effluent were measured daily at the time of feedbag exchange. From d 9 through d 13, effluent (5 mL) was preserved with 1 mL of 25% metaphosphoric acid (w/v) for analysis of volatile fatty acid (VFA). Another subsample of effluent (5 mL) was preserved with 1 mL of 1% H_2_SO_4_ (vol/vol) for NH_3_-N analysis. All samples were stored in frozen at − 20 °C until analysis. Concentration of VFA was quantified using a gas chromatograph (model 5890, Hewlett-Packard Lab, Palo Alto, CA, USA) equipped with a capillary column (30 m × 0.32 mm i.d., 1-μm phase thickness, Zebron ZB-FAAP, Phenomenex, Torrance, CA, USA) and flame ionization detection, with crotonic acid (trans-2-butenoic acid) used as internal standard. The concentrations (mmol/L) of total VFA were calibrated based on daily effluent volume (L/d) to determine daily production of total VFA. The NH_3_-N concentration was determined as described by Rhine et al. [23]. For protozoa enumeration, 1 mL of liquid was gently squeezed from the 48-h incubated feed bag and transferred to a screw-cap vial containing 1 mL of methyl green-formalin-saline solution on d 11 to 13. The samples were stored at room temperature and prevented from light until counting by light microscopy with a Levy-Hausser counting chamber (Hausser Scientific, Horsham, PA, USA).

Total gas production was measured daily using a gas meter (Model DM3A, Alexander-Wright, London, UK). From d 9 to 13, a volume of 20 mL gas was collected from each gas collection bag using a syringe into evacuated 6.8 mL-exetainers (Labco Ltd., Wycombe, Bucks, UK). Methane concentration were determined using a Varian 4900 gas chromatograph equipped with a GS-Carbon PLOT 30 m × 0.32 mm × 3 μm column and thermal conductivity detector (Agilent Technologies Canada Inc., Mississauga, ON, Canada) at an isothermal oven temperature of 35 °C, with helium as the carrier gas (27 cm/s).

### Microbial protein synthesis

Microbial protein synthesis was estimated as the sum of microbial biomass in the form of liquid-associated bacteria (LAB), feed particle-associated (FPA) and feed particle-bound (FPB) bacterial fractions. On d 14 and 15, the daily total effluent for each fermenter was measured and a subsample (250 mL) was centrifuged (20,000×*g*, 30 min, 4 °C) for isolation of LAB. The resulting pellets were washed with PBS and centrifuged (20,000×*g*, 30 min, 4 °C) three times prior to suspension in distilled water, and stored in frozen. at the meantime, feedbags followed by 48 h of incubation, were put in a specific plastic bag with 20 mL of McDougall’s buffer and processed for 1 min in a Stomacher 400 Laboratory Blender (Seward Medical Ltd., London, UK). Feed residues were hand washed twice with 10 mL of McDougall’s buffer. All processed liquid was collected in centrifuge tubes, centrifuged (500×*g*, 10 min, 4 °C) to remove large feed particles, and the supernatant was centrifuged (20,000×*g*, 30 min, 4 °C) following the same procedure as for the LAB for obtaining FPA pellet. Washed feed residues (FPB fraction) were dried at 55 °C for 48 h and weighed for amount of solid DM determination. The LAB and FPA pellets were freeze-dried and the samples (LAB, FPA and FPB) were ball ground (MM400; Retsch Inc., Newtown, PA, USA) for analysis of N and ^15^N by combustion analysis coupled to a mass spectrometer (NA1500, Carlo Erba Instruments, Milan, Italy).

### Microbial community

Each of FPA and LAB sample were pooled from d 14 and 15 collection, and total microbial DNA was extracted using the Qiagen DNeasy PowerLyzer PowerSoil kit (Qiagen Inc., Mississauga, ON, Canada) according to manufacturer’s instructions. The concentration of DNA was measured using the Qubit dsDNA BR Assay Kit (Thermo Fisher Scientific Inc., Waltham, MA, USA) with a Qubit 2.0 Fluorometer (Thermo Fisher Scientific Inc., Waltham, MA, USA). Negative extraction controls were included in duplicate for control of extraction contamination. The extracted DNA was stored at − 20 °C until sequencing.

All PCR amplification and sequencing steps were carried out at Genome Quebec (Montreal, QC, Canada). Libraries of 16S rRNA gene sequence were generated using a two-step PCR protocol. The V4 region of the 16S rRNA gene was amplified using the universal bacterial and archaeal primers 515-F (GTGYCAGCMGCCGCGGTAA) and 806-R (GGACTACNVGGGTWTCTAAT) in the first step of PCR [24]. A unique 10-bp barcode and Illumina (Illumina, San Diego, CA, USA) adapter sequences were added at the 5′ end of each amplicon in the second PCR step. The 16S rRNA gene amplicons were quantified using a Quant-iT PicoGreen dsDNA assay kit (Invitrogen, Burlington, ON, Canada), pooled in equimolar ratios, and then purified with AMPure XP beads (Beckman Coulter, Mississauga, ON, Canada). Sequencing of 16S rRNA gene amplicons was carried out according to manufacturer’s instructions using an Illumina MiSeq (2 × 250) and the MiSeq Reagent Kit v2 (500 cycles; Illumina).

Sequencing quality was checked with FastQC 0.11.5 and MultiQC 1.0 [25]. De-noised reads were used to construct amplicon sequence variants (SVs) using QIIME2 [26]. Analysis of 16S rRNA gene sequences was processed and analyzed within the QIIME2 and the R package DADA2 (Version 1.4). Sequences were then assigned to operational taxonomic units (OTUs) at 97% similarity using an open-reference OTU picking method and the Greengenes database (v13_8). In this method, sequences that were less than 97% similar to those in the Greengenes database were clustered into OTUs using the de novo approach and USEARCH. The Shannon diversity index and observed OTU were calculated in QIIME2 and Bray-Curtis dissimilarities were assessed using the R packages vegan v. 2.4.4 [27] and phyloseq v. 1.20.0 [28]. Alpha diversity was estimated by observed OTUs and Shannon diversity index by using QIIME2. Beta diversity was performed to evaluate differences in overall bacterial communities by non-metric multidimensional scaling (NMDS), based on Bray-Curtis dissimilarities, using the vegan package of the R software suite. The significance of between-groups differentiation on Bray-Curtis dissimilarity was assessed by PERMANOVA using the adonis function of the R package vegan with 999 permutations. Differentially abundant OTUs between treated group and control group were identified with a threshold of 5% using DESeq2 [29].

### Statistical analysis

Data were analyzed using the MIXED procedure of SAS (SAS Inc., Cary, NC, USA). The model included the fixed effects of treatment, day and treatment × day interactions with the day of sampling from each fermenter treated as a repeated measure with individual fermenter considered as the experimental unit. The minimum values of Akaike’s Information Criterion was used to select the covariance structure among compound symmetry, heterogeneous compound symmetry, autoregressive, heterogeneous autoregressive, unstructured and banded for each parameter. The effect of day and its interactions were removed from the model when we had just 1 day of sampling or when different day samples were combined for analysis. The protozoa count data were normalized by log10 transformation prior to statistical analysis. Data were tested for normality of variance. Orthogonal polynomial contrasts were performed to test for linear and quadratic responses to SRU at different addition level (i.e., control, U28, U56). Contrasts were generated to compare the control and MU35. The differences were declared significant at *P* ≤ 0.05 and trends at 0.05 < *P* ≤ 0.10.

## Results

### Effects of SRU on nutrient disappearance and gas production

Disappearance of DM, OM, CP, NDF, ADF and starch was not affected by increasing the SRU supplementation in the diets (Table 2). However, the disappearance of DM (*P* < 0.01), OM (*P* < 0.03) and CP (*P* < 0.04) was greater with MU35 than control diets. Gas production (L/d) tended (*P* < 0.07) to be quadratically changed with increasing SRU supplementation, but it increased compared to control in U28 and decreased in U56, and it was also lower (*P* < 0.04) with MU35 than control. Increasing dietary SRU addition tended (*P* < 0.08) to quadratically changed methane production, expressed as mg CH_4_/g digested DM.

**Table 2:** Effect of slow-release urea (SRU) supplementation on nutrient disappearances and gas production in RUSITEC

Item	Treatment^a^					*P*^b^		
	Control	U28	U56	MU35	SEM	L	Q	C vs. MU35
Digestibility, %								
DM	74.1	74.9	74.9	78.5	0.96	0.51	0.66	0.01
OM	74.6	74.6	74.1	78.9	1.03	0.74	0.86	0.03
CP	75.3	77.8	76.2	81.2	1.58	0.73	0.35	0.04
NDF	45.7	43.4	45.3	51.9	2.46	0.93	0.56	0.10
ADF	41.7	41.0	39.1	47.2	2.44	0.49	0.88	0.12
Starch	98.6	98.5	97.9	98.4	1.11	0.18	0.10	0.23
Gas production, L/d	1.79	1.92	1.67	1.51	0.09	0.31	0.07	0.04
CH_4_, % of gas	4.22	4.34	3.85	4.34	0.39	0.30	0.32	0.81
CH_4_, mg/d	45.43	55.27	43.18	44.92	4.25	0.70	0.05	0.86
CH_4_, mg/g digested DM	6.18	7.18	5.64	5.60	0.55	0.51	0.08	0.42

^a^Control (no SRU), U28 = control plus 0.28% SRU, U56 = control plus 0.56% SRU, and MU35 = control diet was modified by partially replacing soybean protein with 0.35% SRU (DM basis);

^b^L, Q = linear and quadratic effects of SRU (control, U28 and U56); C vs. MU35 = Contrast between Control and MU35

### Effects of SRU on media pH and fermentation characteristics

Fermenter pH was slightly lower (*P* < 0.03) with MU35 than control but it was not affected with increasing the dose of SRU (Table 3). Total VFA concentration or daily VFA production did not differ with increasing SRU supplementation, but it tended (*P* < 0.08) to be greater with MU35 than control diet. The increase of dietary SRU addition did not change molar proportion of acetate and butyrate, but linearly (*P* < 0.01) decreased the proportion of propionate, and quadratically changed the proportion of branch-chained VFA (*P* < 0.01), valerate (*P* < 0.03) and caproate (*P* < 0.01), and linearly (*P* < 0.01) increased acetate to propionate ratio. There was lower (*P* < 0.04) proportion of butyrate and greater (*P* < 0.02) branch-chained VFA as well as greater (*P* < 0.02) acetate to propionate ratio with MU35 than control diet. Concentration of NH_3_-N either linearly (*P* < 0.01) increased with increasing SRU supplementation or it was greater (*P* < 0.01) with MU35 than control, whereas, protozoa counts in fermentation media did not differ among treatments.

**Table 3:** Effect of slow-release urea supplementation on fermentation pH and characteristics in Rusitec

Item	Treatment^a^					*P*^b^		
	Control	U28	U56	MU35	SEM	L	Q	C vs. MU35
pH	6.81	6.80	6.80	6.77	0.01	0.25	0.47	0.03
NH_3_-N, mmol/L	7.70	9.31	10.25	8.92	0.37	0.01	0.39	0.03
Total VFA, mmol/L	78.5	80.2	79.3	85.7	2.86	0.21	0.65	0.08
% of total VFA								
Acetate (A)	46.1	45.6	46.4	47.2	0.57	0.57	0.16	0.16
Propionate (P)	26.9	25.7	26.0	26.5	0.21	0.01	0.01	0.16
Butyrate	14.9	15.5	15.2	14.2	0.24	0.38	0.13	0.04
BCVFA^3^	2.8	3.2	2.9	3.2	0.09	0.87	0.01	0.02
Valerate	7.8	8.4	7.9	7.4	0.25	0.63	0.03	0.22
Caproate	1.3	1.6	1.5	1.4	0.05	0.02	0.01	0.08
A:P	1.69	1.77	1.78	1.77	0.03	0.01	0.24	0.02
Total VFA, mmol/d	50.5	53.1	52.0	55.9	2.05	0.13	0.18	0.06
Protozoa, × 10^4^/mL	2.4	2.9	2.8	2.8	0.24	0.91	0.17	0.24

^a^Control (no SRU), U28 = control plus 0.28% SRU, U56 = control plus 0.56% SRU, and MU35 = control diet was modified by partially replacing soybean protein with 0.35% SRU (DM basis);

^b^L, Q = linear and quadratic effects of SRU (control, U28 and U56); C vs. MU35 = Contrast between Control and MU35

^c^BCVFA = Branched-chain volatile fatty acids (isobutyrate + isovalerate)

### Effects of SRU on microbial protein synthesis and microbial community

Increasing SRU addition increased total microbial N production (quadratic, *P* = 0.05), and the production of FPB (linear, *P* < 0.06), and linearly (*P* < 0.03) improved microbial N efficiency (Table 4). There were greater (*P* < 0.03) production of total microbial N and trend (*P* < 0.08) of greater FPB production with MU35 than control diet, but the differences in LAB and FPA production, and the microbial N efficiency were not significant between MU35 and control group.

**Table 4:** Effect of slow-release urea (SRU) supplementation on microbial N production in Rusitec

Item	Treatment^a^					*P*^b^		
	Control	U28	U56	MU35	SEM	L	Q	C vs. MU35
Microbial N^c^, mg/d								
Total	78.9	84.5	83.8	83.4	1.88	0.14	0.05	0.03
LAB	52.8	56.0	55.6	55.3	1.69	0.38	0.61	0.20
FPA	15.0	15.7	14.9	15.3	0.81	0.97	0.47	0.79
FPB	9.8	11.4	11.5	11.5	0.65	0.06	0.32	0.08
Efficiency of microbial N^d^	9.5	11.5	11.7	10.2	0.30	0.03	0.12	0.17

^a^Control (no SRU), U28 = control plus 0.28% SRU, U56 = control plus 0.56% SRU, and MU35 = control diet was modified by partially replacing soybean protein with 0.35% SRU (DM basis);

^b^L, Q = linear and quadratic effects of SRU (control, U28 and U56); C vs. MU35 = Contrast between Control and MU35

^c^LAB = liquid associate bacteria; FPA = feed particle-associated bacteria; FPB = feed particle-bound bacteria

^d^Efficiency of microbial N, mg of microbial N production/g of OM fermented

**Fig. 1:**
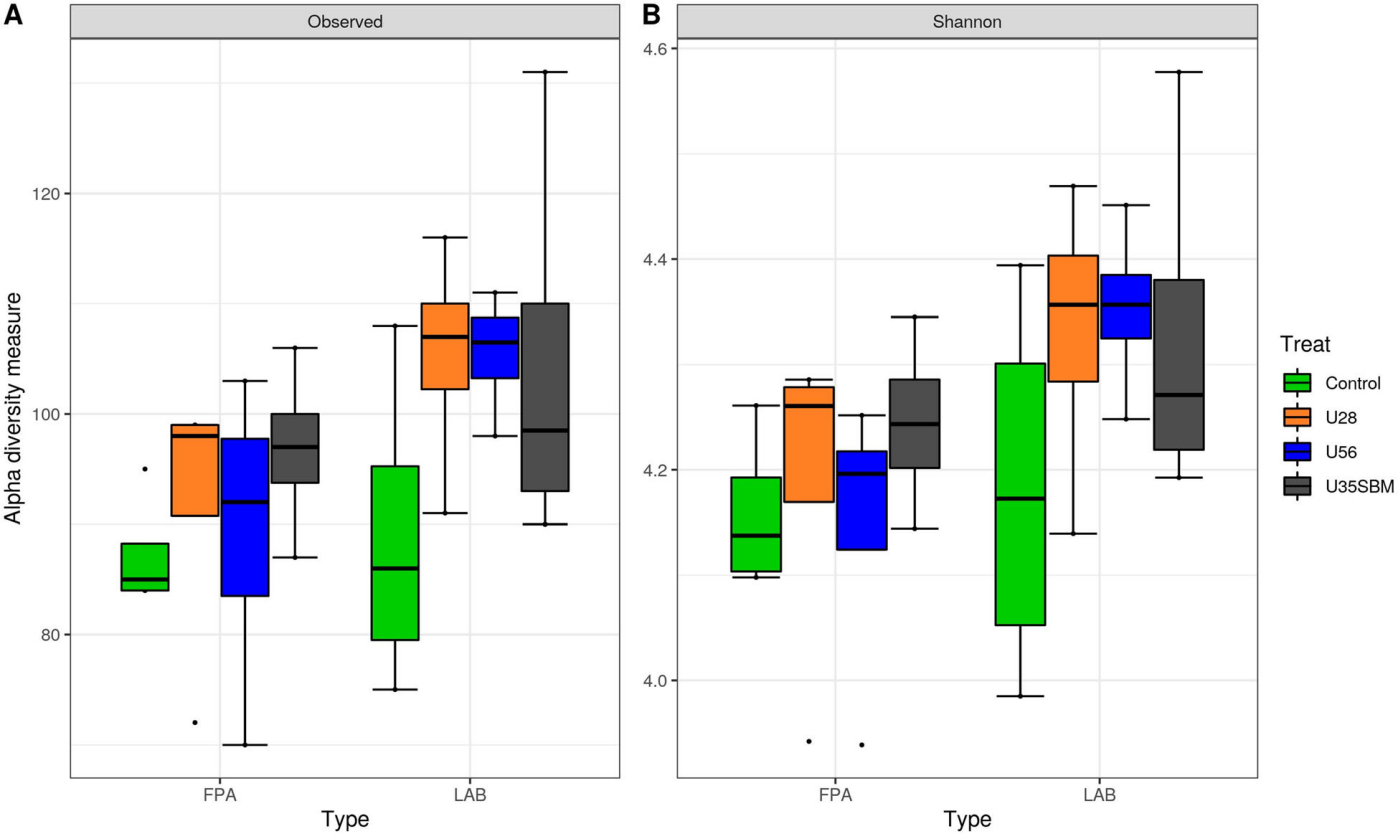
Box plots of the (**A**) observed OTUs and (**B**) Shannon diversity index for feed particle-associated (FPA, left) and liquid-associated bacteria (LAB, right) samples by treatment. Treatments were: control diet (no SRU), U28 = control plus 0.28% SRU, U56 = control plus 0.56% SRU, and MU35 = control diet was modified by partially replacing soybean protein with 0.35% SRU (DM basis). Different lowercase letters within each sampling time represent different means (*P* < 0.05). Error bars indicate ± standard error of the mean. The box in the plots indicates the interquartile range (IQR) (middle 50% of the data), the middle line represents the median value, and the whiskers represents 1.5 times the IQR

**Fig. 2:**
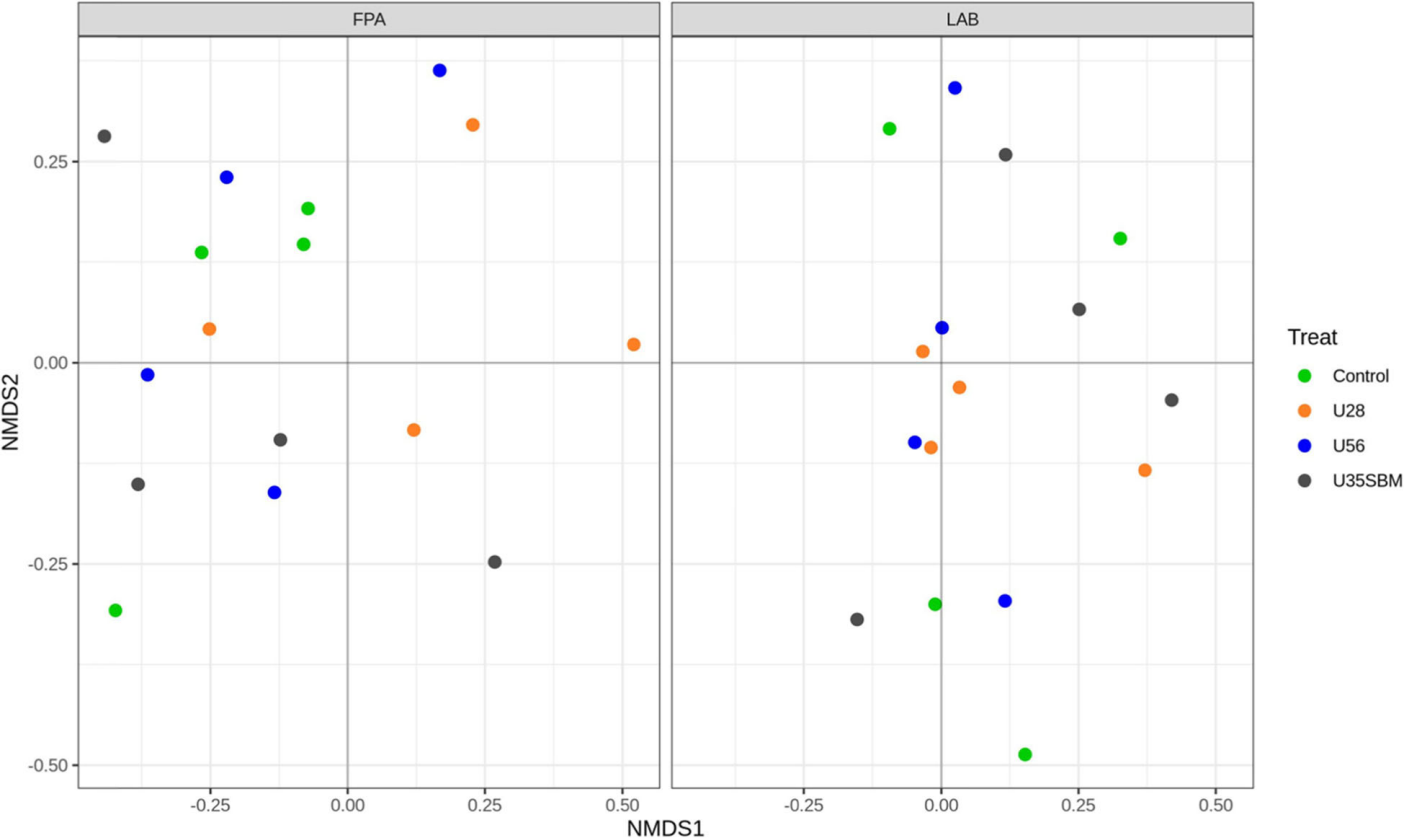
Non-metric multidimensional scaling (NMDS) plots of the Bray-Curtis dissimilarities for feed particle-associated (FPA, left) and liquid-associated bacteria (LAB, right) samples by treatment. Treatments were: control diet (no SRU), U28 = control plus 0.28% SRU, U56 = control plus 0.56% SRU, and MU35 = control diet was modified by partially replacing soybean protein with 0.35% SRU (DM basis)

**Fig. 3:**
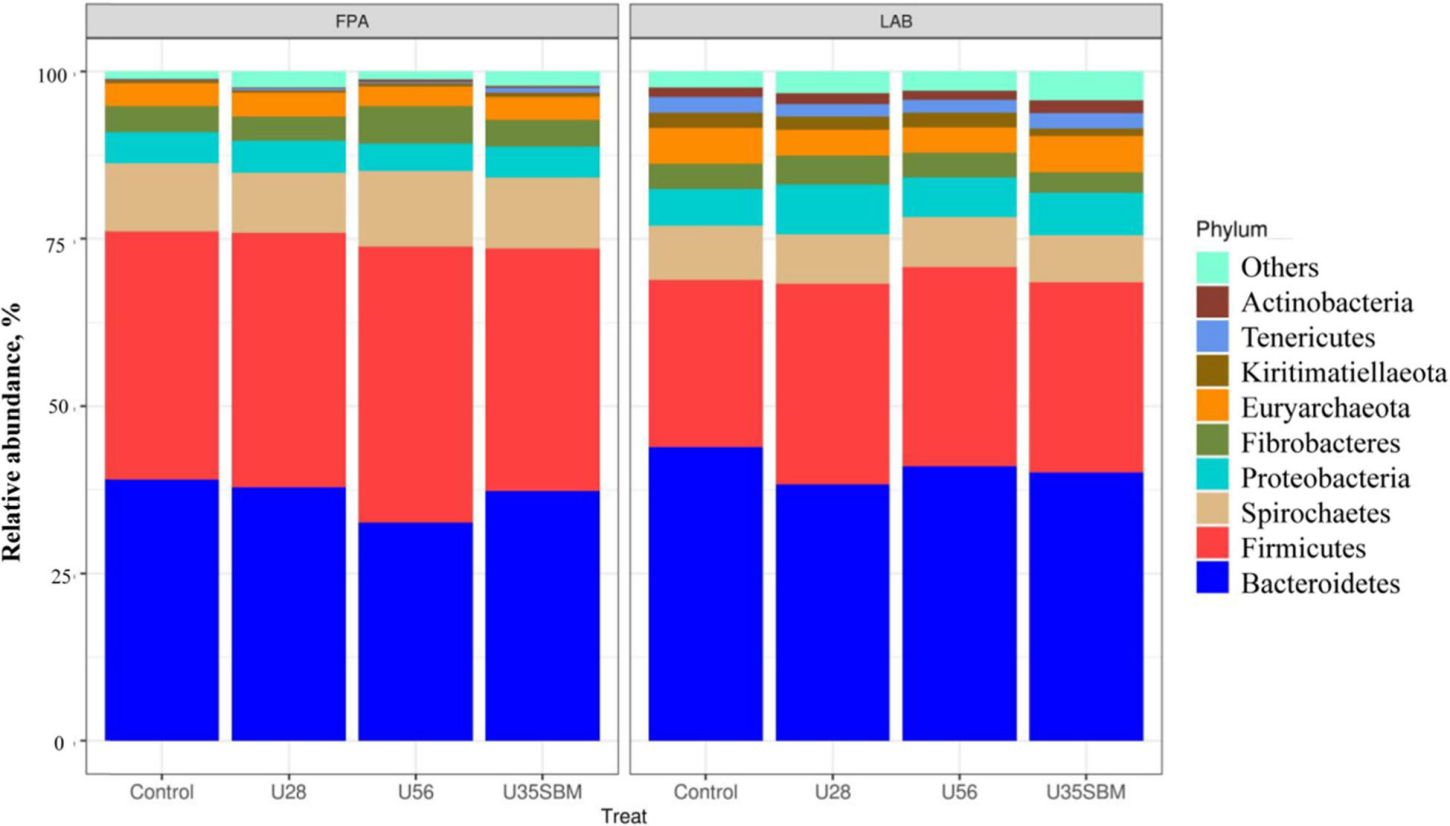
Effects of slow-release urea (SRU) on the relative abundance of ruminal microbial at phylum level for feed particle-associated (FPA, left) and liquid-associated bacteria (LAB, right) samples. Treatments were: control diet (no SRU), U28 = control plus 0.28% SRU, U56 = control plus 0.56% SRU, and MU35 = control diet was modified by partially replacing soybean protein with 0.35% SRU (DM basis)

**Fig. 4:**
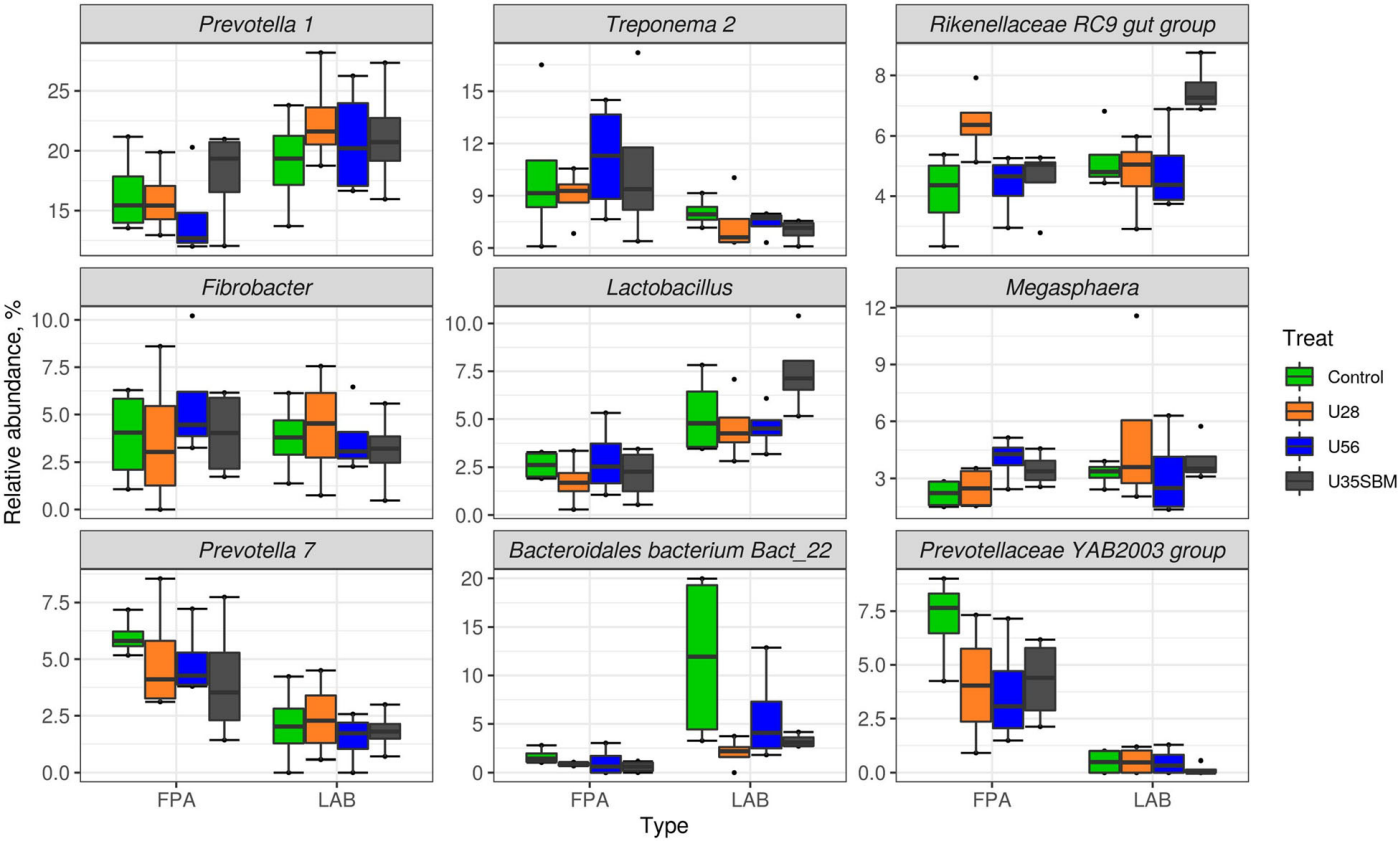
Relative abundance of the 9 most abundant genus in the microbiota of FPA and LAB samples by treatment. Treatments were: control diet (no SRU), U28 = control plus 0.28% SRU, U56 = control plus 0.56% SRU, and MU35 = control diet was modified by partially replacing soybean protein with 0.35% SRU (DM basis)

The FPA and LAB samples were selected for high-throughput sequencing to assess the microbial community in the present study. Neither observed OTUs (Fig. 1A) nor Shannon diversity index (Fig. 1B) of FPA and LAB microbial community were affected by SRU supplementation. Similarly, the results of the NMDS indicated that there was no specific clustering of FPA and LAB microbial community either by adding SRU or by substituting SRU for SBM (Fig. 2). At the phylum level (Fig. 3), Bacteroidetes (38.6%), Firmicutes (33.2%), Spirochaetes (8.8%), Proteobacteria (5.5%), Fibrobacteres (4.1%), Euryarchaeota (4.0%), were the predominant (> 94% of total), and they were overall not different among treatments except for Firmicutes. The RA of Firmicutes tended (*P* = 0.06) linearly increase with increasing SRU supplementation, and it was greater with MU35 than control in LAB samples. At the genus level (Fig. 4), *Prevotella* 1 (18.6%), *Treponema* 2 (8.8%), *Rikenellaceae RC*9 *gut group* (5.2%), *Fibrobacter* (4.1%), *Lactobacillus* (3.9%), *Megasphaera* (3.4%), *Prevotella* 7 (3.4%), *Bacteroidales bacterium Bact*_22 (3.2%) and *Prevotellaceae YAB*2003 *group* (2.5%) were considered as the “core bacteria” in FPA and LAB. For FPA samples. The RA of *Megasphaera* linearly (*P* < 0.04) increased and the RA of *Rikenellaceae RC*9 *gut group* quadratically (*P* < 0.05) changed with increasing SRU addition in FPA sample. *Prevotellaceae YAB*2003 *group* in LAB samples tended (*P* = 0.06) linearly increased with increasing SRU supplementation. In comparison with control, the MU35 had greater RA of *Rikenellaceae RC*9 *gut group* in LAB (*P* = 0.01) and *Prevotellaceae YAB*2003 *group* in FPA (*P* = 0.07).

**Fig. 5:**
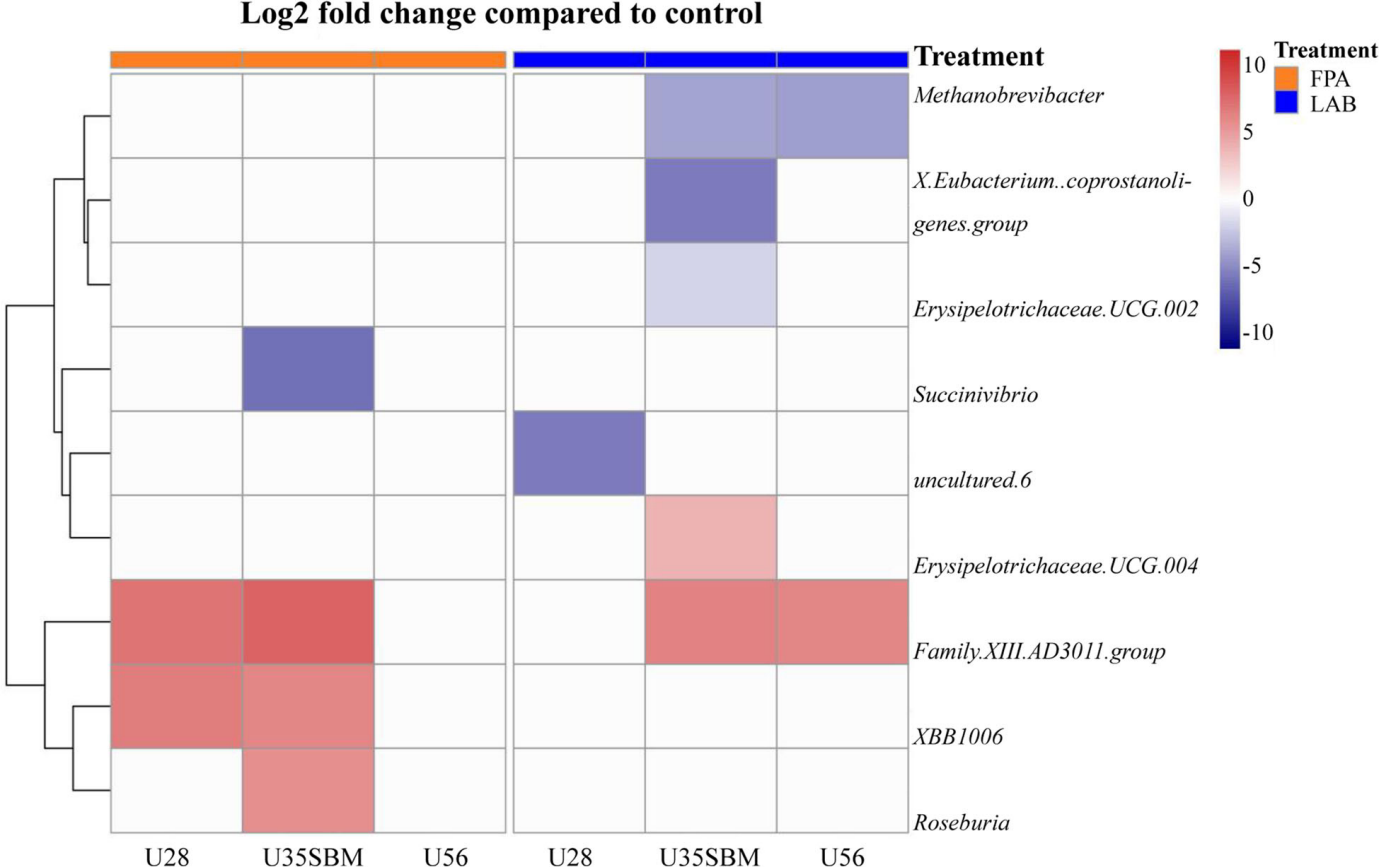
Relative changes (log2 fold change; *P*_adj_ < 0.05) of ruminal bacterial by treatment vs. control (CON) at genus level, orange for feed particle-associated (FPA, left) samples and blue for liquid-associated bacteria (LAB, right) samples. Treatments were: control diet (no SRU), U28 = control plus 0.28% SRU, U56 = control plus 0.56% SRU, and MU35 = control diet was modified by partially replacing soybean protein with 0.35% SRU (DM basis)

The Log2 fold change analysis of the FPA samples found that several genera from phyla Firmicutes and Proteobacteria had 5% more change (enhanced or reduced; *P*_adj_ < 0.05) with SRU than the control (Fig. 5). The U28 group had more (*P*_adj_ < 0.05) *Family XIII AD*3011 *group* and *XBB*1006 from phyla Firmicutes than control. The MU35 treatment enhanced (*P*_adj_ < 0.05) the abundance of *Roseburia* from phyla Firmicutes, but reduced (*P*_adj_ < 0.05) the abundance of *Succinivibrio* from phyla Proteobacteria as compared to the control. Similarly, genus of *Family XIII AD*3011 *group* from phyla Firmicutes was also susceptible to SRU supplementation in LAB samples; it was enhanced (*P*_adj_ < 0.05) with U56 and MU35 diets compared with control and U28. Whereas, RA of *Methanobrevibacter* from phylum Euryarchaeota of LAB sample decreased (*P*_adj_ < 0.05) with U56 and MU35. The MU35 versus control also had lower (*P*_adj_ < 0.05) *Eubacterium coprostanoligenes* and *Erysipelotrichaceae UCG*-002, and higher (*P*_adj_ < 0.05) *Erysipelotrichaceae UCG*-004.

## Discussion

### Effects of SRU on nutrient disappearance and gas production

The absent effect of increasing the dose of SRU in the diets from 0, 0.28% to 0.56% on nutrient digestibility is in agreement with some studies [7, 30, 31], but it is consistent with other studies [9, 32]. With using continuous culture system, Xin et al. [7] found no differences in the nutrient digestibility of corn-based dairy cow diet supplemented with either feed grade urea or polyurethane coated urea. Gardinal et al. [31] reported that the nutrient digestibility in the total digestive tract was not affected by adding 2% polymer-coated SRU in the diet of Nellore steers. In contrast, Alipour et al. [9] reported quadratically increased the digestibility of NDF and ADF of a high concentrate diet with increasing SRU dosages from 0, 0.5%, 1.0% to 1.75% in high concentrate diet in Rusitec. Another study has been demonstrated to increase total tract DM and CP digestibility when fed polymer-coated SRU to lactating dairy cows [6]. The theory behind using SRU is sustained release of urea within rumen to have a more consistent supply of ammonia N for increasing fibrolytic microbial activity and the microbial protein synthesis. It is clear that ammonia N concentration can influence fiber digestion in the rumen, but the literature has disagreements on the optimal ammonia N concentration for fiber digestion (19 and 23 mg/dL with Mehrez et al. [33]; 15 and 30 mg/dL with Leng and Nolan [34]). Bryant reported that ammonia N concentration higher than 5 mg/dL is essential for the growth of cellulolytic bacteria [35]. Whereas, Alipour et al. [9] speculated that each diet has an optimal ammonia concentration because the microbial protein synthesis and ammonia use relate to the rate and extent of carbohydrate fermentation. In the present study, the lack of SRU effect (U28 and U56 diets) on the nutrient digestibility might be due to the low dose of SRU supplementation, and the differences in the ammonia N concentration was relatively small among treatments. Similarly, under the comparable experimental conditions to the current experiment (i.e., dairy diet, continuous culture and 0.6% SRU), Xin et al. [7] also did not observed the significant differences in the nutrient digestibility between feed grade urea and SRU diets.

The greater digestibility (+ 5.9%) of DM and OM with MU35 than control was resulted from increased the digestibility of CP (+ 7.8%) and NDF (+ 13.6%). The higher digestibility of DM, CP and NDF is consistent, respectively, with greater total VFA production, higher acetate to propionate ratio and increased microbial protein production with MU35 versus control. However, the improved nutrient digestibility with MU35 compared with control is a bit surprised, because the diet formulation and nutrient contents were overall similar between two treatments. Slightly higher steam-flaked corn at the expenses of SBM in MU35 may provide more digestible energy in the rumen and potentially increase microbial activity and microbial protein synthesis. This speculation can be explained by the higher CP degradability and numerically greater fibre digestibility.

The quadratic changes of gas production with increasing SRU supplementation may be explained by the linear increased NH_3_-N concentration, especially for U56 diet. Ammonia neutralizes the VFA and lower CO_2_ liberation from buffered fermentation media and, as a result, decrease gas production [36]. In comparison of MU35 with control, although more OM was fermented and greater VFA was produced, gas production (L/d) was lower. The lower total gas production along with the higher digestibility of OM with MU35 indicated an improved fermentation efficient. With in-vitro rumen fermentation, truly digested substrates are divided among VFA, gas and microbial biomass, thus the lower gas production was explained by greater VFA and microbial mass production with MU35. Lack of the treatment effect on CH_4_ concentration and production could be due to the same forage to concentrate ratios among diets. Fermentation of forage usually generates more CH_4_ compared with that of concentrate [37].

### Effects of SRU on fermentation characteristics

Fermenter pH (averaged 6.80) was much higher than the rumen pH commonly reported in lactating dairy cow; the high pH was due to well buffered media by continuous buffer infusion with relative small amount of substrate incubated (10 g/d). Although increasing the addition of SRU quadratically changed the molar proportion of propionate, BCVFA, valerate and caproate, their differences among treatments were quantitatively minor. The linearly increased the ratio of acetate to propionate with increasing SRU suggests that the fermentation pattern switched to more acetate production. However, the increased the ratio of acetate to propionate was primarily resulted from a linear reduction of propionate proportion rather than increased acetate production, that is consistent with the lack of difference in NDF digestibility. The linear reduction of propionate proportion also partly explained the quadratically increased methane production with increasing SRU supplementation. The effects of dietary supplementation of SRU on rumen VFA concentration and individual VFA profiles in literature are inconsistent; for example, no difference in total or individual VFA concentrations in Rustitec [7], a decrease in the molar proportion of acetate [38], whereas an increase in the production of acetate were reported [9]. It suggests that the influence of SRU on rumen VFA production vary with diet, dose of SRU, and the methodology.

The lower fermenter pH with MU35 than control is consistent with the higher total VFA concentration and greater OM digestibility. The greater ratio of acetate to propionate with MU35 than control is the result of its numerically higher NDF digestibility. A high acetate to propionate ratio is an indication of proportionally higher digestible NDF in the feeds. Additionally, the higher BCVFA proportion in MU35 group can be explained the increased protein degradation. Formation of BCVFA occurs during protein degradation due to branched chain amino acid deamination [39].

### Effects of SRU on microbial protein synthesis and microbial community

Ruminal microbial protein synthesis is primarily driven by ruminal availability of energy and protein. The greater total microbial protein production with MU35 than control was resulted from numerically increased FPB and LAB fractions, and it is accordance with the higher OM digestibility. However, the quadratically increased microbial protein synthesis with increasing SRU addition from 0, 0.28% to 0.56% was not supported by the OM digestibility, which was not different among the three doses of SRU. Whereas, the linearly increased ammonia N concentration with increasing SRU addition maybe beneficial to microbial protein production in the present study. Xin et al. [7] measured in vitro rumen ammonia N release dynamics, and suggested that adding SRU in dairy cow diets would prolong microbial utilization of additional N sources during ruminal fermentation. Therefore, improve the synchronization between ruminal NH_3_-N release and carbohydrate availability, consequently result in greater microbial protein synthesis. The results observed from studies using beef steers [31, 40] support the suggestion that addition of SRU made a greater synchronization of fermentable carbohydrates and N in rumen.

Rumen microbial diversity and community composition based on next generation sequencing were affected by adding SRU in yak diet [41]. However, in the present study, the microbial community observed as OTUs or Shannon diversity index in both FPA and LAB samples only numerically increased with SRU supplementation compared to control because of substantial individual variation. Similarly, the results of the NMDS indicated no specific clustering of microbial community from both FPA and LAB fractions. In contrast, a study on rumen bacteria of finishing bull found that low level of urea (0.8%) addition had a higher Shannon index and observed OTUs than high level of urea group (2%). This discrepancy may be caused by the low dose of SRU in the current study (max. 0.56%). In fact, the supplementation rate of SRU was 1% and 2%, respectively, for the low and high levels added to yak diet in the study of Yan et al. [8], of which the diversity index (Shannon index) for the low SRU group was higher than that for the high SRU group. It suggests that the impact of SRU addition on rumen microbial community is a dose-dependent. Spanghero et al. [36] reported that the high ammonia N concentration can inhibit rumen bacterial growth. The SRU reduced ammonia N concentration through the inhibition of the hyper-ammonia-producing bacteria, a small group of ruminal bacteria that are responsible for the most of the NH_3_-N production [10].

Firmicutes and Bacteroidetes were the top 2 phyla, which was consistent with the results of previous study on rumen of yak [8], beef cattle [41], and bull [1]. The greater RA of Firmicutes with MU35 than control in LAB samples is consistent with the increased digestibility of OM, VFA production and microbial protein synthesis. Min et al. [42] reported that a higher phylum Firmicutes populations was related to lower A:P ratio and higher ADG, and the authors suggested that the great abundance of Firmicutes within the rumen may play a role in improving feed efficiency. Additionally, the MU35 reduced the RA of *Succinivibrio* from phyla Proteobacteria compared with control. The RA of *Succinivibrio* was positively correlated with butyrate and valerate, indicating *Succinivibrio* was a main contributor for higher concentrations of butyrate and valerate [43]. This would explain the lower concentration of butyrate with MU35 group than control.

The enhanced genus of *Family XIII AD*3011 from phyla Firmicutes in U56 and MU35 indicated its susceptible to SRU supplementation in LAB samples. The information and function of genus *Family XIII AD*3011 *group* in the rumen is limited. *Methanobrevibacter* is able to use hydrogen and carbon dioxide, and format as substrates for methane production. Our results showed that the RA of *Methanobrevibacter* from phylum Euryarchaeota was lower with U56 than control for LAB fraction, which is consistent with quadratic change of CH_4_ production, expressed as mg/d or mg/g digested DM.

## Conclusion

Increasing supplementation of dairy cow diet with SRU from 0, 0.28% to 0.56% in DM basis linearly increased fermenter NH_3_-N concentration, ratio of acetate to propionate and microbial protein efficiency without affecting nutrient digestibility in RUSITEC. These results suggested that maintaining high NH_3_-N concentration due to adding SRU may improve the synchronization between ruminal NH_3_-N release and carbohydrate availability, consequently result in increase of microbial protein synthesis. Adding SRU along with slightly higher available energy by adding corn grain increased nutrient digestibility, microbial protein synthesis, and decreased gas production. It suggested that increased high fermentable energy such as steam flaked corn may further improve beneficial effect of SRU on the rumen fermentation. Although the small amount of SRU was added in an in vitro fermentation system, the results demonstrated the potential benefits to add the SRU for improving ruminal digestibility and microbial protein synthesis. An in vivo study using dairy cows is warranted to better explore the beneficial impact of SRU.

## Data Availability

The data analyzed during the current study are available from the corresponding author on reasonable request.

## Abbreviations

A:P: Ratio of acetate to propionate
ADF: Acid detergent fibre
BCVFA: Branched-chain volatile fatty acids (isobutyrate + isovalerate)
CP: Crude protein
DM: Dry matter
FPA: Feed particle associated bacteria
FPB: Feed particle-bound bacteria
LAB: Liquid associated bacteria
NDF: Neutral detergent fibre
NMDS: Non-metric multidimensional scaling
NPN: Non-protein nitrogen
OM: Organic matter
RA: Relative abundance
RUSITEC: Rumen simulation technique
SBM: Soybean meal
SRU: Slow-release urea
TMR: Total mixed ration
VFA: Volatile fatty acid
